# Isolation and Biological Characterization of Homoisoflavanoids and the Alkylamide *N*-*p*-Coumaroyltyramine from *Crinum biflorum* Rottb., an Amaryllidaceae Species Collected in Senegal

**DOI:** 10.3390/biom11091298

**Published:** 2021-08-31

**Authors:** Marco Masi, Manoj Koirala, Antonella Delicato, Roberta Di Lecce, Natacha Merindol, Seydou Ka, Matar Seck, Angela Tuzi, Isabel Desgagne-Penix, Viola Calabrò, Antonio Evidente

**Affiliations:** 1 Dipartimento di Scienze Chimiche, Universita’ di Napoli Federico II, Complesso Universitario Monte Sant’Angelo, Via Cintia 4, 80126 Napoli, Italy; roberta.dilecce@unina.it (R.D.L.); tuzi@unina.it (A.T.); evidente@unina.it (A.E.); 2 Département de Chimie, Biochimie et Physique, Université du Québec à Trois-Rivières, 3351, Boul. des Forges, C.P. 500, Trois-Rivières, QC G8Z 4M3, Canada; manoj.koirala@uqtr.ca (M.K.); natacha.merindol@uqtr.ca (N.M.); seydou.ka@uqtr.ca (S.K.); isabel.desgagne-penix@uqtr.ca (I.D.-P.); 3 Dipartimento di Biologia, Universita’ di Napoli Federico II, Complesso Universitario Monte Sant’Angelo, Via Cintia 4, 80126 Napoli, Italy; antonella.delicato@unina.it; 4 Laboratoire de Chimie Organique et Chimie Thérapeutique, Faculté de Médecine, de Pharmacie et d’Odontologie de Dakar, Dakar, Senegal; matarsec@yahoo.fr

**Keywords:** *Crinum biflorum*, homoisoflavanoids, alkylamides, cytotoxicity, antioxidant, antidiabetic and anti-acetylcholinesterase activities

## Abstract

*Crinum biflorum* Rottb. (syn. *Crinum distichum*) is an Amaryllidaceae plant used in African traditional medicine but very few studies have been performed on this species from a chemical and applicative point of view. Bulbs of *C. biflorum*, collected in Senegal, were extracted with ethanol by Soxhlet and the corresponding organic extract was purified using chromatographic methods. The pure compounds were chemically characterized by spectroscopic techniques (1D and 2D ^1^H and ^13^C NMR, HR MS and ECD) and X-ray analysis. Four homoisoflavonoids (**1**–**4**) and one alkylamide (**5**) were isolated and characterized as 5,6,7-trimethoxy-3-(4-hydroxybenzyl)chroman-4-one (**1**), as 3-hydroxy-5,6,7-trimethoxy-3-(4-hydroxybenzyl)chroman-4-one (**2**), as 3-hydroxy-5,6,7-trimethoxy-3-(4-methoxybenzyl)chroman-4-one (**3**) and as 5,6,7-trimethoxy-3-(4-methoxybenzyl)chroman-4-one (**4**), and the alkylamide as (*E*)-*N*-(4-hydroxyphenethyl)-3-(4-hydroxyphenyl)acrylamide (**5**), commonly named *N*-*p*-coumaroyltyramine. The relative configuration of compound **1** was verified thanks to the X-ray analysis which also allowed us to confirm its racemic nature. The absolute configurations of compounds **2** and **3** were assigned by comparing their ECD spectra with those previously reported for urgineanins A and B. Flavanoids **1**, **3** and **4** showed promising anticancer properties being cytotoxic at low micromolar concentrations towards HeLa and A431 human cancer cell lines. The *N*-*p*-coumaroyltyramine (**5**) was selectively toxic to A431 and HeLa cancer cells while it protected immortalized HaCaT cells against oxidative stress induced by hydrogen peroxide. Compounds **1**–**4** also inhibited acetylcholinesterase activity with compound **3** being the most potent. The anti-amylase and the strong anti-glucosidase activity of compound **5** were confirmed. Our results show that *C. biflorum* produces compounds of therapeutic interest with anti-diabetic, anti-tumoral and anti-acetylcholinesterase properties.

## 1. Introduction

Plants and microorganisms are well-known sources of bioactive metabolites which have only been partly investigated [1]. Among the plants’ kingdom, the Amaryllidaceae is a plant family extensively studied essentially for its alkaloids and related isocarbostiryls content which show a broad spectrum of biological activities [2,3,4,5]. These plants are principally diffused in tropical and subtropical regions of the world, as Andean South America, the Mediterranean basin and Southern Africa [6], and include ca 1600 species classified into about 75 genera [7]. Hundreds of Amaryllidaceae alkaloids with different structures and biological activities were isolated and reported in several reviews. A Special Issue of the journal *Molecules* was edited by Bastida J. and Berkov S. in 2020 on different aspects of Amaryllidaceae alkaloids including biodiversity, chemoecology, pharmacology, in vitro production, structural characterization, mass spectrometry and molecular modeling [8]. Advances on the chemical and biological characterization of the alkaloids and analogs isolated in the last decade from this plant family were reviewed by Masi et al. (2020) [9] and embedded in this Special Issue [8].

*Crinum* is a well-known subgroup of Amaryllidaceae studied for a long time and shown to be very rich in crinine-type alkaloids [10], one of the 12 ring-type in which are grouped the Amaryllidaceae alkaloids [11]. Three undescribed alkaloids, gigantelline, gigantellinine and gigancrinine with anti-acetylcholinesterase activity, were recently isolated from *Crinum jagus*, together with the already known lycorine-, cherylline-, galanthamine- and crinine-type alkaloids [12]. We also showed that *C. jagus* crude alkaloid extract inhibited Dengue virus (DENV) infection [13]. Among the alkaloids isolated from this plant, cherylline inhibited efficiently of both DENV (EC_50_ = 8.8 μM) and Zika virus (ZIKV) replication (EC_50_ = 20.3 μM), whereas it was ineffective on human immunodeficiency virus type 1 (HIV-1) infection. Thus, cherylline could be optimized for new therapeutic approaches against flaviviruses [13].

Amaryllidaceae plants, and in particular the subfamily Amaryllidoideae, also produce metabolites belonging to other classes of natural compounds such as flavonoids, lignans, chromones, terpenoids and ceramides. However, metabolites different from alkaloids are less studied. Still, up to now about 223 compounds, essentially flavan and phytosterols, were isolated from Amayllidoideae, all with interesting biological activities and taxonomical importance [14]. In particular, flavonoids are a well-known class of natural compounds possessing protective action against oxidative stress, heart disease and some cancers, and are considered healthy for humans and livestock [15,16].

Examples of non-alkaloids bioactive metabolites used in folk medicine include 2(*S*)-4′-hydroxy-7-methoxyflavan with cytotoxic activity [17] and vanillin [18] from *Crinum bulbispermum*, while amabiloside and its 4-glucoside was isolated from *Crinum amabile* [19]. *Crinum latifolium*, a rare species growing in Vietnam, produced a coumarin derivative as 4-[(senecioyloxy)methyl)]-6,7-dimethoxycoumarin showing strong antiangiogenic activity [20]. *Crinum aurea* produced (7*S*)-7-(4-hydroxyphenyl)-7-hydroxypropyl)-2′-methylbenzene-3′,6′-diol exhibiting neuroprotection against H_2_O_2_/CoCl_2_-induced neuronal cell death in dopaminergic neuroblastoma SH-SY5Y cells [21]. *Crinum asiaticum* L. var. *sinicum* produced several benzoic and cinnamic acid derivatives [22]. *Crinum purpurascens* produced β-sitosterol showing a weak antibiotic activity [23] while *Crinum ensifolium* produced parthenicin, a sesquiterpene exhibiting strong cytotoxic activity [24]. *Crinum angustum* produced aliphatic hydroxylketones [25] while *Crinum yemense* produced 6-hydroxy-2*H*-pyran-3-carbahaldehyde showing strong inhibition of tyrosinase enzyme [26]. Acetovanillone and 4-hydroxyacetophenone, also known, respectively, as apocynin and piceol, were recently isolated from *Crinum buphonoides*, while only the former was isolated from *Crinum graminicola* [27].

*Crinum biflorum* Rottb. (syn. *Crinum distichum*) is found in seasonally flooded places in savannas from Senegal to Nigeria and extending to Sudan [28] but few studies have been carried out on its constituents and their biological properties [29].

Thus, the present study aimed to identify compounds with interesting biological activities from bulbs of *C. biflorum* collected in Senegal. Four homoisoflavonoids and one alkylamide were isolated. The chemical and biological properties were characterized to envisage their potential nutraceutical and pharmacological applications. This study expands the library of compounds isolated from *C. biflorum* and shows for the first time that this Amaryllidaceae species produces compounds of therapeutic interest against diabetes, cancer and Alzheimer’s disease.

## 2. Materials and Methods

### 2.1. General Experimental Procedures

A JASCO P-1010 digital polarimeter (Jasco, Tokyo, Japan) was used to measure the optical rotations in CH_3_OH. Electronic circular dichroism (ECD) spectra were recorded on a JASCO J-815 spectrometer (Jasco, Tokyo, Japan) in CH_3_OH. 400 Anova Advance (Bruker, Karlsruhe, Germany) and Inova 500 MHz (Varian, Palo Alto, CA, USA) instruments were used to record ^1^H and ^13^C NMR spectra in CDCl_3_ or CD_3_OD at 400/100 or 500/125 MHz, respectively. The same solvents were used as internal standards. Correlation spectroscopy with a 45° mixing pulse (COSY-45), Nuclear Overhauser effect spectroscopy (NOESY), heteronuclear single quantum correlation (HSQC) and heteronuclear multiple bond correlation (HMBC) experiments were performed using Bruker or Varian microprograms. Electrospray ionization (ESI) mass spectra and liquid chromatography/mass spectrometry (LC/MS) analyses were carried out using the LC/MS time-of-flight (TOF) system Agilent 6230B, high-performance liquid chromatography (HPLC) 1260 Infinity. A Phenomenex LUNA [C_18_ (2) 5 um 150 × 4.6 mm column] was utilized to perform the HPLC separations. Analytical and preparative thin-layer chromatography (TLC) were performed on silica gel (Kieselgel 60, F_254_, 0.25 and 0.5 mm, respectively, Merck) plates. The spots were visualized by exposure to ultraviolet (UV) radiation (254) or iodine vapors. Column chromatography (CC) was performed using silica gel (Kieselgel 60, 0.063–0.200 mm, Merck). Sigma-Aldrich Co. (St. Louis, MO, USA) supplied all the reagents and the solvents.

### 2.2. Plant Material

Bulbs of *C. biflorum* were collected in Senegal, in Kaffrine department, in December 2018. A senior scientist from the Herbarium of IFAN of University Cheikh Anta Diop of Dakar taxonomically identified the plant materials.

### 2.3. Extraction and Purification of Compounds ***1**–**5***

Fresh bulbs of *C. biflorum* were dried at room temperature and then finely powdered. The resultant powder (545 g) was extracted with ethanol by Soxhlet obtaining a semisolid brown extract (450 mg). The latter was fractionated by CC eluted with CHCl_3_/EtOAc/CH_3_OH (3:1.5:0.5 *v*/*v*/*v*), affording eight groups of homogeneous fractions (F1-F8). The residue (102.8 mg) of fraction F1 was further purified by CC eluted with CHCl_3_/*iso*PrOH (97:3 *v*/*v*) yielding ten groups of homogeneous fractions (F1.1-F1.10). The residue (15.8 mg) of F1.3 was purified by preparative TLC eluting with CH_2_Cl_2_/MeOH (97:3 *v*/*v*) yielding compound **4** (3.04 mg). The residue (14.3 mg) of F1.5 was purified by preparative TLC eluting with *n*-hexane/EtOAc (6:4 *v*/*v*) yielding compound **1** (1.42 mg) and compound **3** (3.52 mg). Compound **1** was crystallized from a CHCl_3_-*iso*PrOH 9:1 *v*/*v* solution. The residue (6.7 mg) of F1.6 was purified by TLC eluted with CHCl_3_/*iso*PrOH (9:1 *v*/*v*) yielding compound **2** (1.61 mg). The residue (15.2 mg) of F4 was further purified by preparative TLC eluting with CHCl_3_/*iso*PrOH (95:5 *v*/*v*) yielding compound **5** (1.19 mg).

### 2.4. Spectroscopic Data of Compounds ***1**–**5***

5,6,7-trimethoxy-3-(4-hydroxybenzyl)chroman-4-one (**1**). [α]^25^_D_: 0 (*c* 0.3 CH_3_OH); ^1^H NMR data are very similar to those reported by Sylao et al. 1999 [30]; ESIMS (+) *m*/*z*: 727 [2M + K]^+^, 719 [2M + Na]^+^, 383 [M + K]^+^, 367 [M + Na]^+^, 345 [M + H]^+^.

3-hydroxy-5,6,7-trimethoxy-3-(4-hydroxybenzyl)chroman-4-one (**2**). ECD (*c* 0.010 mM, CH_3_OH) ∆ε 255 (−1.1), 280 (+2.5), 317 (−1.6); lit. [31]: ECD (*c* 0.031 mM, CH_3_OH) ∆ε 281 (+10.4), 316 (−7.3); ^1^H NMR data are very similar to those reported for urgineanin B [31]; ESIMS (+) *m*/*z*: 383 [M + Na]^+^, 361 [M + H]^+^.

3-hydroxy-5,6,7-trimethoxy-3-(4-methoxybenzyl)chroman-4-one (**3**). ECD (*c* 0.010 mM, CH_3_OH) ∆ε 255 (−1.1), 281 (+2.5), 317 (−1.6); lit. [31]: ECD (c 0.031 mM, CH_3_OH) ∆ε 280 (+10.8), 315 (−7.2); ^1^H NMR data are very similar to those reported for ungirneanin A [31]; ESIMS (+) *m*/*z*: 397 [M + Na]^+^, 375 [M + H]^+^.

5,6,7-trimethoxy-3-(4-methoxybenzyl)chroman-4-one (**4**). [α]^25^_D_: 0 (*c* 0.2 CH_3_OH); ^1^H NMR data are very similar to those reported by Sidwell and Tamm (1970) [32]; ESIMS (+) *m*/*z*: 381 [M + Na]^+^, 359 [M + H]^+^.

(*E*)-*N*-(4-hydroxyphenethyl)-3-(4-hydroxyphenyl)acrylamide (*N*-*p*-coumaroyltyramine) (**5**). ^1^H and ^13^C NMR data are very similar to those reported by Bhatti et al. (1992) [33]; ESIMS (+) *m*/*z:* 306 [M + Na]^+^; ESIMS (−) *m*/*z*: 282 [M − H]^−^.

### 2.5. Crystal Structure Determination of Compound ***1***

Single crystals of **1** suitable for X-ray structure analysis were obtained by slow evaporation of a CHCl_3_-*iso*PrOH 9:1 *v*/*v* solution. One selected crystal was mounted at ambient temperature on a Bruker–Nonius KappaCCD diffractometer (Bruker-Nonius, Delft, The Netherlands) (graphite monochromated MoKα radiation, λ = 0.710 73 Å, CCD rotation images, thick slices and φ and ω scan to fill the asymmetric unit). A semi-empirical absorption correction (multiscan, SADABS) was applied. The structure was solved by direct methods using the SIR97 program [34] and anisotropically refined by the full-matrix least-squares method on F^2^ against all independently measured reflections using the SHELXL-2018/3 program [35] with the aid of program WinGX [36]. Water solvent crystallization molecules are present in the structure. The hydroxy and water H atoms were located in different Fourier maps and freely refined with Uiso(H) equal to 1.2 Ueq of the carrier atom. All of the other hydrogen atoms were introduced in calculated positions and refined according to the riding model with C−H distances in the range of 0.93−0.96 Å and with Uiso(H) equal to 1.2 Ueq or 1.5 Ueq (Cmethyl) of the carrier atom. One stereogenic center is present in the compound that crystallizes in the centrosymmetric P-1 space group as a racemate. The E-statistics indicate that the structure is centrosymmetric. Two independent X-ray structure analyses performed on different crystals confirmed the result. Unitary cell parameters were checked on several crystals. Figures were generated using ORTEP-3 [36] and Mercury-CSD-3.9. [37]. Crystallographic data of 1: empirical formula: C_19_H_20_O_6_·H_2_O; formula weight: 362.36 g mol^−1^; triclinic, P-1; a: 8.368(2) Å; b: 10.183(2) Å; c: 12.0420(6) Å; α: 104.470(8)°; β: 108.252(13)°; γ: 100.010(19)°; V: 907.4(3) Å^3^; Z: 2, Dx: 1.326 Mg/m3. All homoisoflavanoid crystallographic data for (**1**) were deposited in the Cambridge Crystallographic Data Centre with deposition number CCDC 2092030. These data can be obtained free of charge from www.ccdc.cam.ac.uk/data_request/cif.

### 2.6. Cell Culture and Reagents

HaCaT, spontaneously immortalized keratinocytes from adult skin, were purchased from Service Cell Line (GmBH, Eppelheim, CLS, Germany) and cultured as described [38,39]. HeLa cervical cancer cells (CCL-2) and A431 (ATCC-CRL1555) human epidermoid carcinoma cells were from American Type Culture Collection (ATCC, Manassas, VA, USA). According to the p53 compendium database (http://p53.fr/tp53-database/the-tp53-cell-line-compendium, accessed on 5 May 2021), HaCaT cells contain mutant p53 (H179Y/R282W), HeLa have p53 impaired function by viral infection while A431 cells contain only one p53 mutated allele (R273H). All mentioned cell lines were cultured in Dulbecco’s Modified Eagle’s Medium (DMEM, Sigma Chemical Co., St. Louis, MO, USA) supplemented with 10% fetal bovine serum (FBS, Hyclone Laboratories, Inc., Logan, UT, USA) at 37 °C in a humified atmosphere of 5% CO_2_. All cell lines were routinely tested for mycoplasma contamination and were not infected.

### 2.7. MTT Assay

Cell viability was assessed by 3-(4,5-dimethylthiazol-2-yl)-2,5-diphenyltetrazolium bromide (MTT) assay following the published procedure [40]. Briefly, cells were seeded at 10^5^/cm^2^ density in 96-well plates. Twenty-four hours later, the medium was changed and supplemented with the specified concentrations of metabolite (from 0.5 to 10 mM in DMSO) for 24 and 48 h. MTT solution 1:10 (stock solution 5 mg/mL) was added to each well and the absorbance was measured in dual-wavelength mode (570 nm and 630 nm). The percentage of cell viability was calculated as follows: mean (A570–A630) and compared to cells supplemented with DMSO alone. Values shown in the plot are mean ± SD of sixfold determinations. Mean and the standard deviation was calculated on biological triplicates using GraphPad Prism8 software (GraphPad, San Diego, CA, USA).

### 2.8. Detection of DNA Damage

Cells were seeded in 35 mm dishes on micro cover glasses (BDH) and treated with the metabolite at a concentration of 10 μM. At 48 h after treatment, cells were washed with cold phosphate-buffered saline (PBS) and fixed with 4% paraformaldehyde Sigma-Aldrich (Merck Life Science, Milan, Italy) for 15 min at RT. Cells were permeabilized with ice-cold 0.5% Triton X-100 for 5 min and then washed with PBS. Cells were then incubated with phospho-histone H2A.X (Ser139) antibody (from Cell Signaling Technologies 9542, Boston, MA, USA) for 1 h, followed by DAPI (Sigma-Aldrich) for 3 min and washed with PBS/0.05% Tween. Coverslip was mounted with Ibidi mounting medium (Ibidi GmbH, Martinsried, Germany). Images were taken with a Zeiss confocal laser-scanning microscope Axio Observer (Zeiss, Ostfilden, Germany) (scale bar, 20 μm). A 40× objective was used and image analysis was performed using Fiji ImageJ open source software project (https://imagej.net/imaging/). All the images were taken with the same setting [39].

### 2.9. Western Blot Analysis

Western blot was performed as previously reported [39,41]. Briefly, 30 μg of whole-cell extracts were separated by sodium dodecyl sulfate polyacrylamide gel electrophoresis (SDS-PAGE), subjected to Western blot and incubated overnight at 4 °C with antibodies. Antibodies against p21WAF, Poly [ADP-ribose] polymerase 1 (PARP1) and actin were from Cell Signaling Technologies 9542, Boston, MA, USA. Each experiment was run in triplicate. Signal intensities of Western blot bands were quantified by Quantity One analysis software (Version Number 2, Biorad Laboratories, London, UK) and analyzed by GraphPad Prism 8.0.2 software (GraphPad, San Diego, CA, USA).

### 2.10. DCFDA Assay

*N*-*p*-coumaroyltyramine antioxidant activity was measured using 2′−7′dichlorofluorescein diacetate (DCFDA), a non-fluorescent compound permeable to the cell membrane, which can be oxidized by reactive oxygen species (ROS) giving a fluorescent compound. Cells were seeded at 2.5 × 10^4^ in 96 well and pre-treated with *N*-*p*-coumaroyltyramine (10 and 100 mM). The medium was removed after 4 h and 1 mM (3%) H_2_O_2_ was added for 45 min, 1.5 and 2.0 h. Cells were washed with PBS and a fresh medium with DCFDA (30 mM) was added for 45 min, then DCFDA was removed by washing in PBS and the cells were harvested. The measurement of ROS was obtained using the Sinergy H4 microplate reader (Gen5 2.07, Thermofisher, Waltham MA, USA). The fluorescence emitted from the cells treated with DCFDA was compared to the untreated cells. Trolox was used as a positive control. Values shown in the plot are mean ± SD of sixfold determinations. The mean and the standard deviation were calculated on biological triplicates using GraphPad Prism 8.0.2 software (GraphPad, San Diego, CA, USA).

### 2.11. 2Pseudotyped HIV-1_GFP_ Infectivity Assay

The anti-HIV-1 activity of compounds **1**–**5** was evaluated using VSV-G pseudotyped NL43_GFP_ infection of human monocytic THP-1 cells. THP-1 and NL4–3_GFP_ were generously provided by Lionel Berthoux and Amita Singh and are described in Ka et al. (2021) [13]. THP-1 cells were seeded at 2.0 × 10^4^ cells per well in 96 well-plates. The next day, cells were treated with 4 concentrations of each compound (12.5, 25, 50 and 100 mM) and then infected with HIV_GFP_ at a MOI of 1. After 72 h, cells were stained with propidium iodide (PI, 0.5 mg/mL) and both PI^+^ and HIV-1_GFP_^+^ infected cells frequencies were assessed on a FC500 MPL cytometer (Beckman Coulter, Inc., Mississauga, ON, Canada) and analyzed using FlowJo software (FlowJo LLC, BD Biosciences, Ashland, OR, USA). Matched concentrations of dimethyl sulfoxide (DMSO) were used as negative controls. All infection assays were performed in triplicate.

### 2.12. α-Glucosidase and α-Amylase Inhibitor Assay

α-glucosidase and α-amylase inhibitor screening kits (colorimetric) were purchased from Biovision (Milpitas, CA, USA). In total, 10 mM of stock solution of all the tested compounds were dissolved in DMSO and serially diluted in the assay buffer of each kit. Experiments were performed according to the manufacturer’s protocol. Briefly, for the α-glucosidase assay, 10 µL of serially diluted compounds at the corresponding concentration (10 nm–1 mM) were added into designated wells of clear 96 well-plates. Subsequently, 10 µL of the α-glycosidase enzyme was added to each well and volume was adjusted to 80 µL and plates were incubated for 15-min at room temperature in dark condition. Then, 20 µL of α-glycosidase substrate mixture was added in all wells and kinetic of reaction was measured at OD: 410 nm for 60 min at 2 min intervals by using a multiplate reader, Biotek instrument, Inc., Canada. Enzyme control (no inhibitor), background control (no enzyme), solvent control (DMSO) and inhibitor control (acarbose) were included in the plates. For the α-amylase assay, 50 µL of serially diluted compounds (3.25 µM to 500 µM) were added into a clear 96-well plate with 50 µL of assay buffer and 50 µL of α-amylase enzymes. The plate was incubated at room temperature in the dark for 10 min. Then 50 µL of the α-amylase substrate was added in all wells. The kinetic of reaction was measured at OD:410 nm for 26 min at intervals of 2 min by using a multiplate reader. Control α-amylase inhibitor was provided by the manufacturer, enzyme control, background control and solvent control were all included. Enzyme inhibition was calculated according to Zhang et al. (2014) [42]. In summary, ODs were plotted according to the time for each sample. Areas under the curve (AUC) were calculated, and enzyme inhibition was measured as 100−(AUC_compound_/AUC_enzyme_) × 100 for each dilution of each compound.

### 2.13. Anti-Acetylcholinesterase Assays

In vitro acetylcholinesterase (AChE) activity was assessed exactly as in Ka et al. (2020) [12] following Ellman’s colorimetric protocol [43] with the Acetylcholinesterase Assay Kit (Abcam Inc., Boston, MA, USA). Briefly, 50 µL serial dilutions (3.9–500 µM) of compounds **1**–**5** were prepared in Tris-HCl pH = 7.9 buffer into designated wells of a clear 96 well-plate. A total of 5 µL of DTNB was added in each well, then 50 µL of diluted acetycholinesterase was added. The plate was incubated for 10 min in the dark. Matched concentrations of DMSO were used as a negative control. Kinetic of reaction was measured in a multiple plate reader at 410 nm in kinetic mode for 40 min at room temperature. The percentage of anti-AChE inhibition was calculated according to the following formula: 100 − [((E − S)/E) × 100], where E is the activity of the enzyme with matched concentrations of DMSO and S is the activity of the enzyme with the test sample.

### 2.14. Statistical Analysis

Statistical analyses were carried out using the GraphPad Prism version 8.1.2 (https://www.graphpad.com/scientific-software/prism/). Data were represented as the mean ± standard deviation and analyzed for statistical significance using ordinary one-way analysis of variance (ANOVA) and multiple comparisons. For all tests, *p* < 0.05 was considered to indicate a statistically significant difference.

## 3. Results

### 3.1. Identification of Metabolites Isolated from C. biflorum

**Figure 1:**
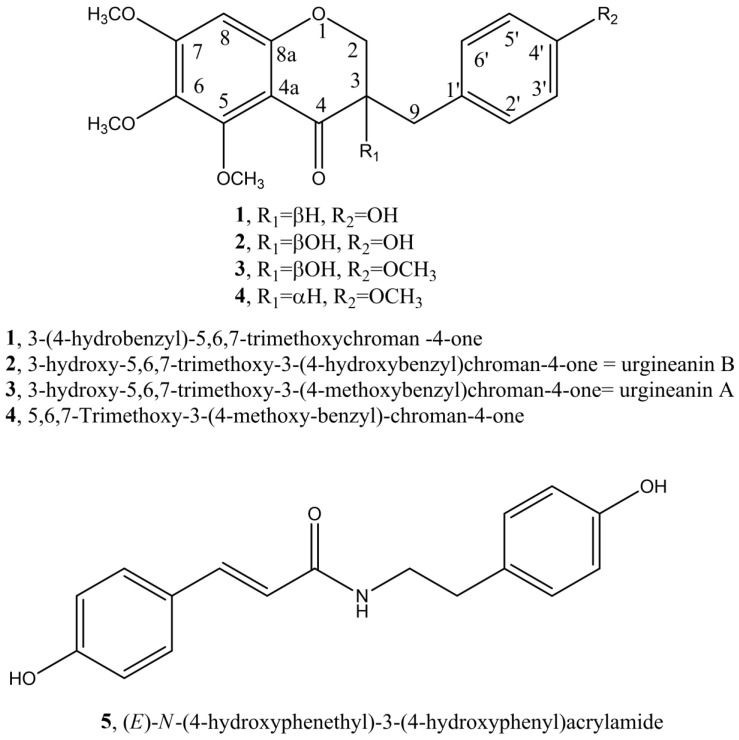
Structure of homoisoflavanoids (**1**)–(**4**) and alkylamide (**5**) isolated from *C. biflorum* bulbs.

The purification of the crude organic extract from bulbs of *C. biflorum* allowed us to isolate four homoisoflavanoids ((**1**)–(**4**), Figure 1) identified using spectroscopic (essentially 1D and 2D ^1^H and ^13^C NMR and HR MS) methods as 5,6,7-trimethoxy-3-(4-hydroxybenzyl)chroman-4-one (**1**), as 3-hydroxy-5,6,7-trimethoxy-3-(4-hydroxybenzyl)chroman-4-one (**2**), as 3-hydroxy-5,6,7-trimethoxy-3-(4-methoxybenzyl)chroman-4-one (**3**) and as 5,6,7-trimethoxy-3-(4-methoxybenzyl)chroman-4-one (**4**).

The identification of **1** was performed comparing its ^1^H NMR spectrum with that reported in the literature when it was isolated for the first time from *Scilla nervosa*, subsp. *rigidifolia* collected in Botswana where it is used in Zulu folk medicine to treat rheumatic fever and as a purge for children [30]. ESI MS spectrum showed the potassium [2M + K]^+^ and sodium [2M + Na]^+^ dimeric, the potassium [M + K]^+^ and sodium [M + Na]^+^ and the protonated [M + H]^+^ adduct ions at *m*/*z*: 727, 711, 383, 367 and 345.

**Figure 2:**
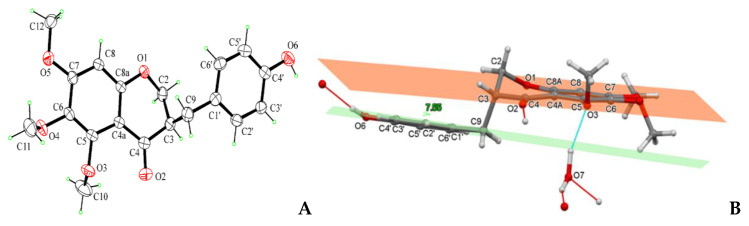
(**A**) ORTEP view of the homoisoflavanoid (**1**) molecular structure, with ellipsoids drawn at the 30% probability level. (**B**) Independent unit of compound 1 in a perspective view showing a quite parallelism between hydroxybenzene plane (green) and benzene ring of chromanone system (orange) (angle of 7.55° between the two planes). Ball-and-stick style. Hydrogen bonds drawn as cyan and red dashed lines.

**Figure 3:**
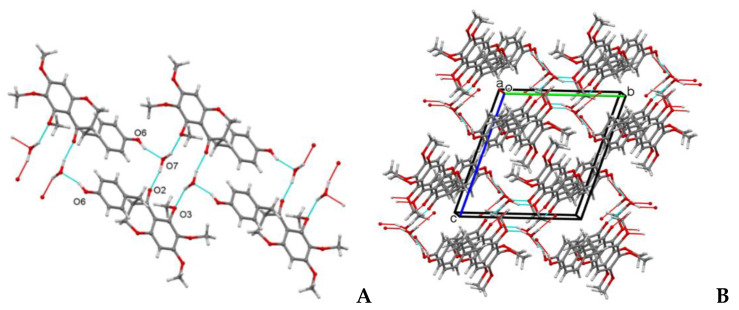
(**A**) Details of hydrogen bonding pattern in compound **1**. (**B**) Perspective view of the crystal packing of compound **1** with hydrogen bond pattern drawn as cyan and red lines. Ball-and-stick style.

The structure of the homoisoflavanoid (**1**) was confirmed by X-ray analysis of a single crystal obtained by slow evaporation of a CHCl_3_-isoPrOH 9:1 *v*/*v* solution. Compound **1** crystallizes in the P-1 space group with one molecule of **1** and one H_2_O solvent molecule contained in the independent unit. All bond lengths and angles are in a normal range. The molecule consists of a substituted cromanone system. The six-membered croman-4-one ring assumes an envelope conformation with C2 atom at the flap. The 4-hydroxybenzyl substituent at C3 is in the equatorial position and places nearly parallel to the benzene ring of the chromanone system (Figure 2A,B). The crystal packing is stabilized by strong OH…O hydrogen bonds involving **1** and solvent water molecules (Figure 3A,B).

**Figure 4:**
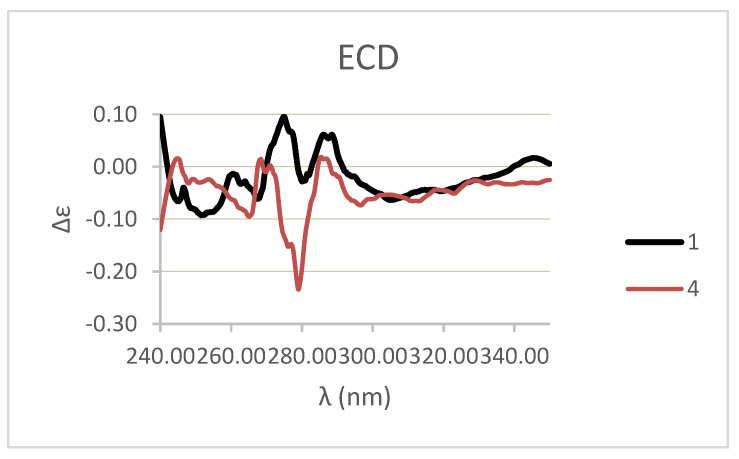
Experimental ECD spectra of homoisoflavanoids (**1**) (black solid line), and **4** (red line).

The homoisoflavonoid (**1**) molecule contains one stereogenic center at C3 atom and crystallizes in the centrosymmetric P-1 space group as a racemate. The racemic nature of crystals was confirmed by performing two independent X-ray structure analyses on different crystals (Figure 2B). This result can be ascribed to easy inversion at the chirality center, because of the presence at C-3 of a proton which could exchange by keto-enol tautomerism. The ECD spectrum of **1** (Figure 4, black line) cannot be interpreted due to the presence of a racemic mixture. These results were confirmed by the optical inactivity found by measuring the specific optical rotation of compound **1**.

**Figure 5:**
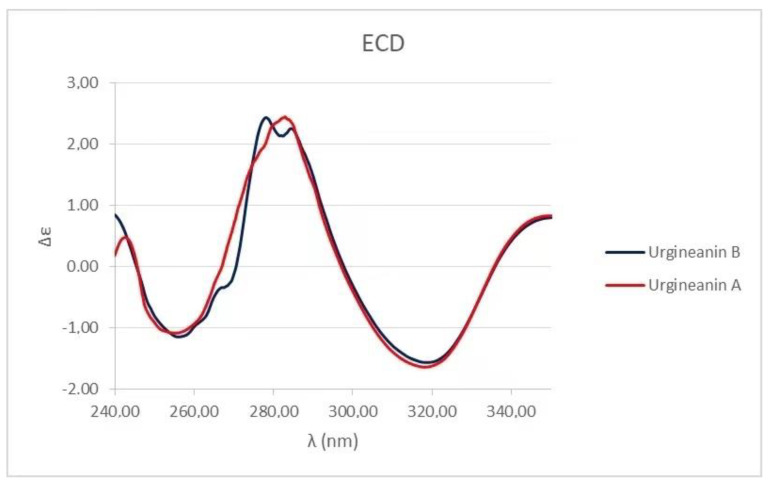
ECD spectra of homoisoflavanoids (**2**) (black solid line), and **3** (red line).

Homoisoflavanoides **2** and **3** were identified by comparing their physic (ECD) and spectroscopic data (^1^H NMR) with those reported in the literature for urgineanins B and A, isolated from *Urginea depressa*. This is an Asparagaceae collected in South Africa and used for its antiproliferative activity against the A2780 ovarian cancer cell line [31]. The ESI MS spectra of both **2** and **3** confirmed their identification showing the sodiated and protonated adduct ions [M + Na]^+^ and [M + H]^+^, respectively, at *m*/*z* 383 and 361 and 397 and 375. The absolute configuration of homoisoflavanoids **2** and **3** was the same of urgineanins B and A as appeared by comparison of their ECD spectra (Figure 5, black line for **2** and red line for **3**) with those already reported in the literature by Dai et al. (2013) [31] which assigned a *R* configuration at C-3 of the two homoisoflavanoids by comparison of their experimental ECD spectra with those reported in literature for caesalpiniaphenol A [31].

The fourth homoisoflavanoid (**4**) was identified as the 5,6,7-trimethoxy-3-(4-methoxybenzyl)chroman-4-one by comparing its physic and spectroscopic data with those reported for the trimethyl derivative of 3,9-dihydroautumnalin obtained together to other homoisoflavanoids from *Eucomis autumnalis* (Liliaceae) [32]. The same derivative was also obtained from 3,9-dihydroeucomnsalin, which resulted to be identical to 3,9-dihydroautumnalin, which was isolated from the bulbs of *Muscari comsum* (commonly named lampascioni) collected in Basilicata region, Italy, where they are used in traditional cuisine as bitter plants [44]. The identification of compound **4** was also supported by ESI MS data which showed the sodiated [M + Na]^+^ and protonated [M + H]^+^ adduct ions at *m*/*z* 381 and 359. The ECD spectrum of **4** (Figure 4, red line) cannot be interpreted, as in the case of compound **1**, due to the presence of a racemic mixture, as confirmed by the optical inactivity found by measuring the specific optical rotation of **4**.

From the ethanolic extract of the *C. biflorum* bulbs and alkylamide was also isolated and identified compound **5**, or (*E*)-*N*-(4-hydroxyphenethyl)-3-(4-hydroxyphenyl)acrylamide (**5**, Figure 1). Compound **5** was identified by comparison of its spectroscopic data with those reported in the literature when isolated from the first time from crude Chinese drug “Xiebai”, the tuber of *Allium bakeri* Reg. (Liliaceae) and used for inhibition on human platelet aggregation [45]. A similar comparison was done with the data reported when **5** was isolated together with some alkaloids from *Fumaria indica*, collected in Multan City (Punjab, Pakistan) and inappropriately indicated as an alkaloid instead of amide [33]. The identification of compound **5** was also supported by ESI MS data which showed the sodiated [M + Na]^+^ adduct ion at *m*/*z* 306. When the same spectrum was recorded in negative modality, the pseudomolecular ion [M − H]^−^ at *m*/*z* 282 was observed.

### 3.2. In Vitro Cytotoxicity

**Figure 6:**
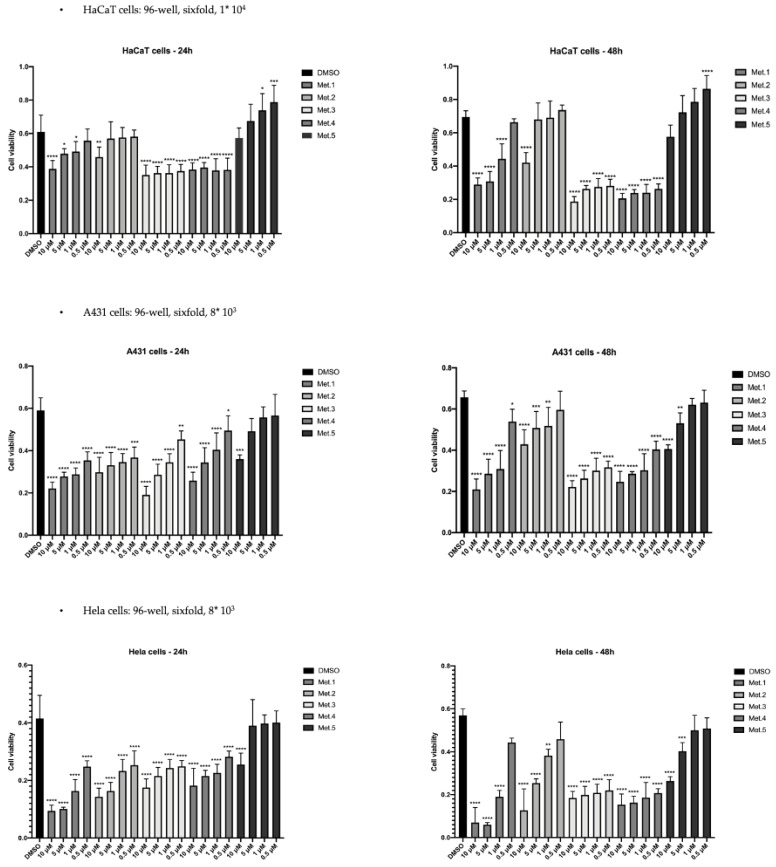
MTT viability assays with homoisoflavanoids **1**–**4** and *N*-*p*-coumaroyltyramine (**5**) from *C. biflorum* on HaCaT, A431 and Hela human cell lines. Statistical analyses were performed using one-way ANOVA and Dunnett’s multiple comparisons test. Levels of significance between points of expression are indicated (**** *p* < 0.001, *** *p* < 0.01, ** *p* < 0.02, * *p* < 0.05).

Considering that the anti-tumor activities of homoisoflavonoids (**1**–**4**) from *C. biflorum* have not yet been addressed, we evaluated the in vitro cytotoxicity of isolated homoisoflavonoids towards HaCaT, A431 and HeLa human cell lines using the MTT viability assay. The *N*-*p*-coumaroyltyramine (**5**) alkylamide was also included in these experiments. Cells were plated and supplemented with each metabolite, at low micromolar concentrations ranging from 0.5 to 10 μM in DMSO, and incubated at 37 °C for 24 and 48 h. As shown in Figure 6, all tested metabolites were toxic for cancer cell lines, in a dose and time-dependent way, with HeLa cells being more sensitive than A431. Remarkably, 48 h treatment with 5 μM metabolite **1** reduced HeLa cells viability to less than 20% of the untreated control. All metabolites were less active on HaCaT immortalized keratinocytes at 24 h of incubation over the range of concentration tested although a significant reduction of HaCaT cell viability was caused by metabolites **3** and **4** when the incubation was extended for 48 h. Table 1 lists the IC_50_ values obtained with the test compounds are means of triplicates at 24 h. The *N*-*p*-coumaroyltyramine (**5**) was also found to be toxic on A431 and HeLa cancer cells while at the lower concentration tested, 0.5 and 1 μM, it was shown to significantly increase HaCaT cell viability (Figure 6). Detection of nuclear γ-H2A.X foci provides indirect evidence of the occurrence of DNA double-strand breaks (DSB) and/or DNA replication stress [40]. Upon induction of a DNA double-strand break, the H2A.X histone becomes rapidly phosphorylated at serine 139 to form γH2AX [46]. This phosphorylation event is dynamic, complex and depends on interactions between MDC1, H2AX and ATM and other kinases to persist [47]. This amplified response is easily detected using specific antibodies against γ-H2AX, manifesting discrete nuclear foci.

**Figure 7:**
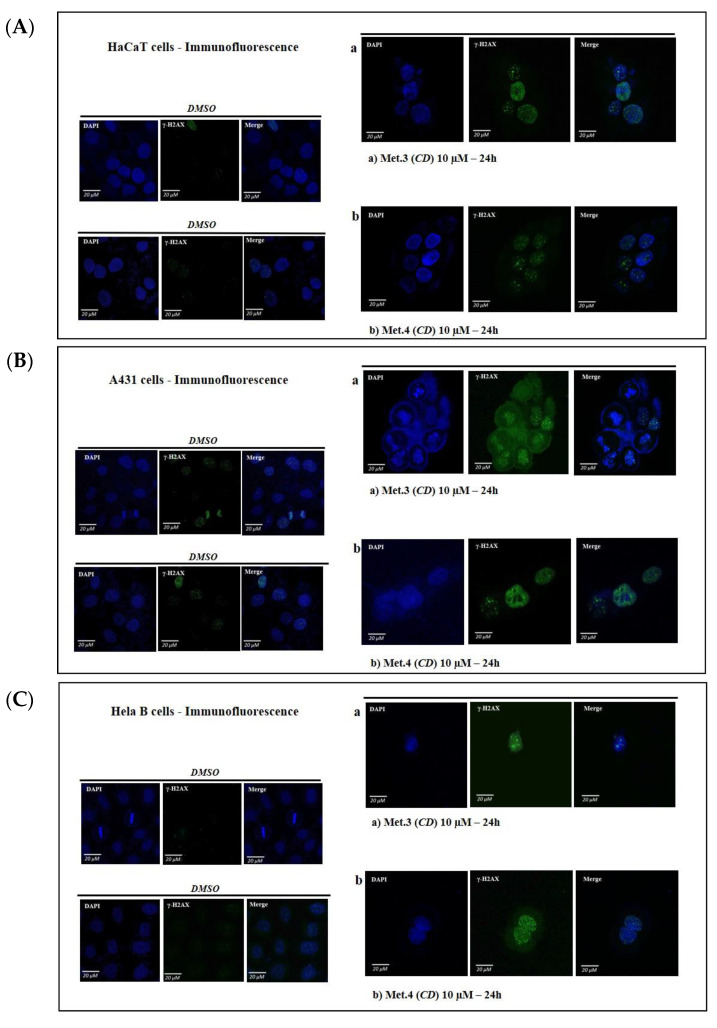

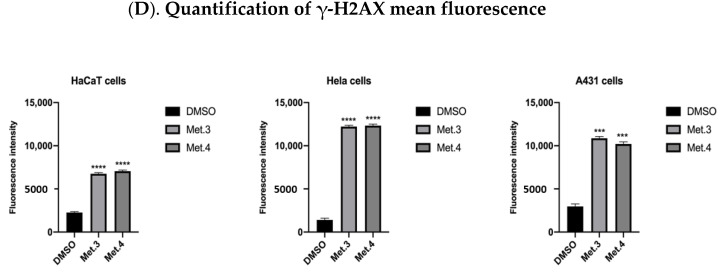
Immunofluorescence microscopy showing γ-H2AX foci formation (green) in nuclei of HaCaT (panel (**A**)), A431 cells (panel (**B**)) or HeLa cells (panel (**C**)) treated with DMSO alone or 10 μM **3** or **4** for 24 h. Nuclei were stained with DAPI (blue). Note the abnormal morphology of nuclei induced by **3** and **4** in A431 cells compared to HaCaT cells. Images from 3 fields per each experimental point were collected to obtain data for up to 150 cells. Quantitation of γ-H2AX foci fluorescence was performed by Image J software shown as mean ± SD in graph bars of panels (**D**). Statistical analyses were performed using one-way ANOVA and Dunnett’s multiple comparisons test. Levels of significance between points of expression are indicated (**** *p* < 0.001, *** *p* < 0.01).

The formation of γ-H2AX foci by immunofluorescence using the antibody against the histone H2A.X phosphorylated in Serine 139 was monitored considering that the homoisoflavonoids isolated from *C. biflorum* reduced cell viability. HaCaT, HeLa and A431 cells were treated with 10 μM metabolites **3** and **4** for 24 h to detect DNA damage foci. As shown in Figure 7 a remarkable increase of nuclear γH2AX foci was observed in all tested cell lines.

**Figure 8:**
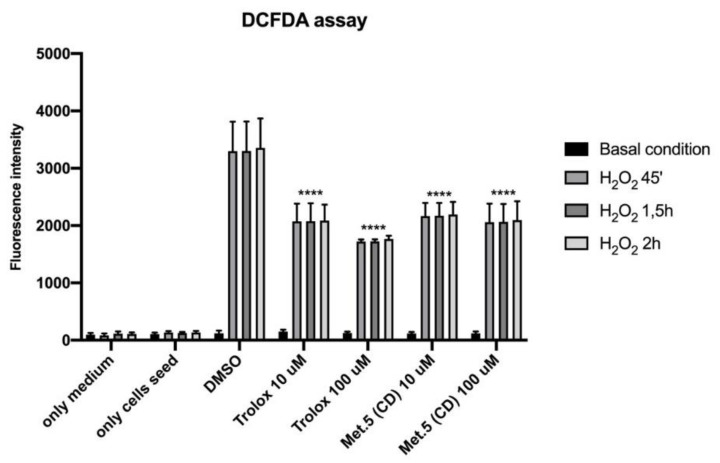
DCFDA assay. HaCaT cells were seeded and pre-treated for 4 h with 10 and 100 μM *N*-*p*-coumaroyltyramine (**5**) from *C. biflorum*. H_2_O_2_ (1 mM; 3%) was added to the medium for 45′, 1.5 and 2 h. The fluorescence intensity of DCFDA was read after 45′ of incubation. Trolox was used as a positive control. Levels of significance between points of expression are indicated (**** *p* < 0.001).

**Figure 9:**
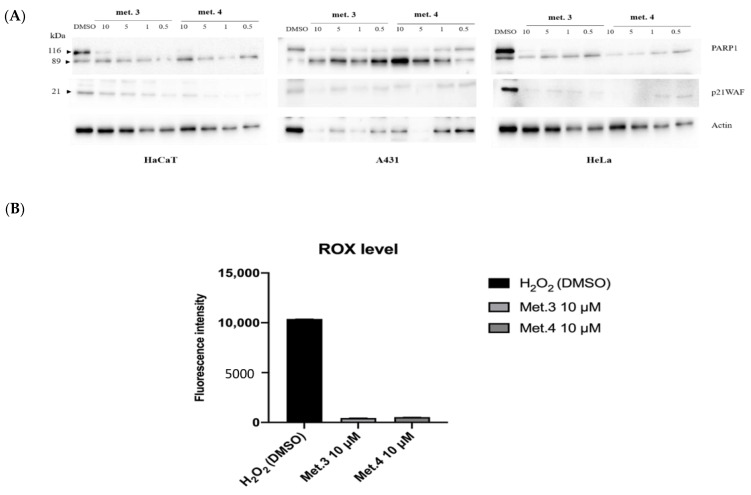
(**A**) Representative immunoblot analysis showing the effect of **3** and **4** on PARP1 activation and p21WAF protein levels. Full length and cleaved PARP1 bands were revealed by immunoblot with specific antibodies. The blots were then re-probed with actin antibody to check for protein loading. (**B**) DCFDA on HaCaT cells pre-treated for 4 h with 10 μM **3** and **4** from *C. biflorum*. H_2_O_2_ (1 mM; 3%) was added to the medium for 45′ and used as a positive control. Fluorescence intensity of DCFDA was read after 45′ of incubation.

Determination of reactive oxygen species (ROS) induced by H_2_O_2_ in HaCaT cells treated with 10 and 100 µM *N*-*p*-coumaroyltyramine alkylamide revealed that **5** has antioxidant activity comparable to Trolox, the water-soluble derivative of vitamin E used as a positive control (Figure 8). Remarkably, treatment of HaCaT cells with 10 µM metabolite **3** or **4** did not increase the production of reactive oxygen species (ROS) implying that ROS generation was not responsible for the observed DNA damage (Figure 9A,B).

### 3.3. Antidiabetic and Anti-Acetylcholinesterase Properties

**Figure 10:**
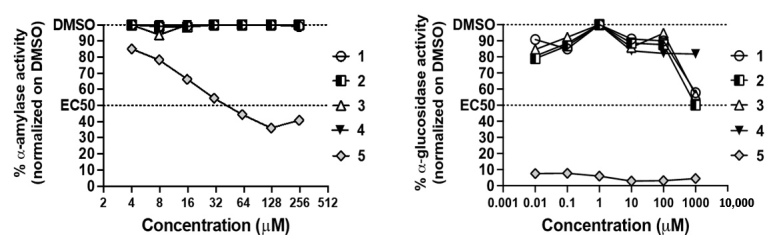
(**A**) Anti-α-amylase activity. Kinetics of α-amylase reaction was measured in presence of each compound at concentrations ranging from 3.9—250 μM, and matched concentrations of DMSO solvent over a 26 min period. Areas under the curves (AUC) of OD (410 nm) vs. time were calculated according to Zhang et al. (2014) [42] and used to determine enzyme activity defined as the ratio of the AUC for each compound at each concentration normalized on the AUC in presence of DMSO matched concentration. Compound **5** strongly inhibited α-amylase catalyzed reaction with an IC_50_ of 40 μM. (**B**) Anti-α-glucosidase activity. Kinetics of α-glucosidase reaction were measured in presence of each compound at concentrations ranging from 0.01–1000 μM, and matched concentrations of DMSO solvent over a 40 min period. Areas under the curves (AUC) of OD (410 nm) vs. time were calculated and used to determine enzyme activity defined as the ratio of the AUC for each compound at each concentration normalized on the AUC in presence of DMSO matched concentration. Compound **5** nearly completely inhibited α-glucosidase catalyzed reaction with at all concentration tested. Compounds **1**, **2** and **3** inhibited 50% of the reaction at 1 mM. For all assays, enzyme, no compound and inhibitor controls were included.

**Figure 11:**
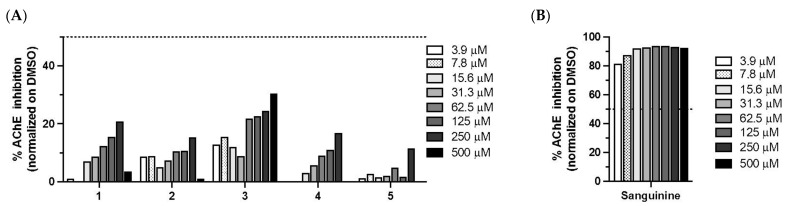
(**A**) Anti-AChE activity. AChE-catalyzed reaction was assessed in presence of each compound at concentrations ranging from 3.9–500 mM and matched concentrations of DMSO after a 10 min incubation. The percentage of anti-AChE inhibition was calculated according to the following formula: 100 − [((E − S)/E) × 100], where E is the activity of the enzyme with matched concentrations of DMSO and S is the activity of the enzyme with the test sample. Compound **5** weakly inhibited AChE catalyzed reaction at high concentrations. Compounds **1**, **2** and **4** displayed increasing inhibition from 15.6 μM to 250 μM up to 20%. Compound **3** was the most potent inhibitor of AChE, blocking 15–30% of enzyme activity as concentrations increased from 3.9 to 500 μM. (**B**) Sanguinine (isolated from *C. jagus* [12]) was used as a positive control. For all assays, enzyme, no compounds and inhibition controls were included.

Next, we assessed the anti-acetycholinesterase and antidiabetic (Figure 10A,B) properties of *C. biflorum*-isolated compounds. We confirmed that compound **5** was a strong inhibitor of α-amylase and α-glucosidase (Figure 10) [48]. Compound **1**, **2**, **3** also inhibited 50% of α-glucosidase at high concentration (1 mM) (Figure 10B). Altogether, the results support the antidiabetic properties of *C. biflorum*. Compounds **1**–**4** also showed anti-acetylcholinesterase activity. Interestingly, compound **5** weakly inhibited AChE catalyzed reaction at high concentrations, while **1**, **2** and **4** displayed increasing inhibition from 15.6 to 250 µM up to 20% (Figure 11). Compound **3** was the most potent inhibitor, blocking from 15 to 30% of enzyme activity as concentrations increased from 3.9 to 50 µM (Figure 11).

### 3.4. Antiviral Activity

**Figure 12:**
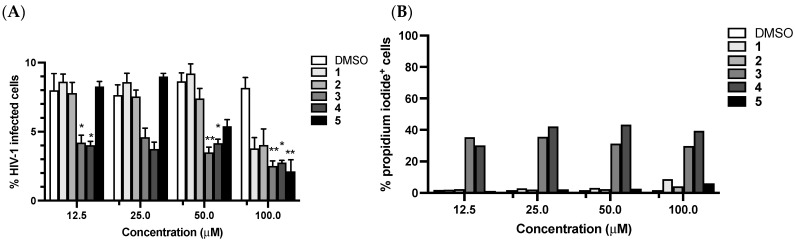
Antiviral activity of compounds **1**–**5.** (**A**) THP-1 cells were treated with four concentrations (12.5, 25, 50 and 100 μM) of each compound in triplicates and infected with VSV-G pseudotyped HIV-1_GFP_ at MOI = 1. Infection levels were assessed by flow cytometry 72 h post-infection. Bars show means with standard deviation. Kruskal–Wallis and uncorrected Dunn’s tests were performed to assess statistically significant differences between groups. * *p* < 0.05; ** *p* < 0.01. (**B**) Cell death (% propidium iodide^+^ cells) was measured by flow cytometry following PI staining 72 h post-treatment of THP-1 with **1**, **2**, **3**, **4** and **5**. Matched concentrations of DMSO were used as a negative control.

Flavonoids and alkylamides have also been shown to exhibit antiviral activity [49]. Thus, we tested the anti-retroviral effect of compounds **1**–**5** using VSV-G pseudotyped HIV-1 particles. Interestingly, all compounds displayed anti-HIV-1 potential at 100 μM (Figure 12A). Infection dropped from a mean of 8.16%, when cells were treated with DMSO, to means of 3.78, 4.03, 2.51, 2.76 and 2.12 %, when compounds **1** to **5** were added, respectively. These differences were statistically significant for compounds **3**, **4** and **5**. At 50 μM, there was a 2.0, 2.4 and 1.6-fold decrease in infection when **3**, **4** and **5** were added to the cell media, respectively. At lower concentrations only **3** and **4** decreased infection levels. Cytotoxicity was verified following propidium iodide staining of THP-1 cells after an incubation of 72 h with compounds (Figure 12B). Compounds **1**, **2** and **5** were weakly cytotoxic to THP-1 at 100 μM with 8.74, 4.15 and 5.97% of PI^+^ cells, respectively. In total, 30–40% of cells treated with **3** and **4** were dead at all concentrations tested. Thus, although cytotoxicity could contribute to the observed antiviral activity, this experiment was performed using a single-cycle infection system, lessening the effect of cell loss on further cycles of viral replication. In conclusion, *C. biflorum* contains compounds with interesting anti-retroviral activity.

## 4. Discussion

Very few studies have been performed on *C. biflorum* (syn. *C. distichum*) from a chemical and applicative point of view. A phytochemical investigation on this Amaryllidaceae species was carried out on the ethanol extract of the whole plant using HPLC analysis. A new natural flavan, (2*S*)-4′,7-dimethoxyflavan, was isolated together with the already known (2*S*)-4′-hydroxy-7-methoxyflavan, (2*R*)-4’-hydroxy-5,7-dimethoxyflavan, hippadine and hippacine. All the compounds, except the new flavan, showed moderate antibacterial activity [50]. Another study was carried out on the ethyl acetate extract from the whole plant of *C. biflorum* collected in Cameroon. Its phytochemical investigation allowed to isolate a new flavan-3-ol derivative, namely (2*R*,3*R*)-3-hydroxy-7-methoxy-3′,4′-methylenedioxyflavan, together with (2*S*)-7-hydroxy-3′,4′-methylenedioxyflavan, (2*R*,3*R*)-7-methoxy-flavan-3-ol, (2*S*)-7-hydroxy-3′,4′-dimethoxyflavan, 3′,7-dihydroxy-4′-methoxyflavan, 4′,7-dimethoxy-3′-hydroxyflavan, farrerol, β-sitosterol, β-sitosterol-3-*O*-β-D-glucopyranoside, oleanolic acid, kaempferol, pancratistatin, lupeol, aurantiamide acetate, narciprimine and 2,3-dihydroxypropyl palmitate [29]. Some of them were also isolated from Muscari species among a series of homoisoflavanones containing the 3-benzylchroman-4-one skeleton [51].

Compounds **2** and **3** were characterized as 3-hydroxy-5,6,7-trimethoxy-3-(4-hydroxybenzyl)chroman-4-one (**2**), as 3-hydroxy-5,6,7-trimethoxy-3-(4-methoxybenzyl)chroman-4-one (**3**) and resulted to be the previously isolated homoisoflavanoids named urgineanins B (**2**) and A (**3**) [31].

No alkaloids were detected in the acid organic extract from bulbs of *C. biflorum*, object of the present study, using either the optimized extraction method [52] or the traditional extraction with ethanol by Soxhlet. However, the organic extract obtained with the latter method showed the presence of four homoisoflavonoids and one alkylamide. The racemate nature of compound **1** was never reported before; only the absolute configuration of its *p*-bromobezoyl derivative was previously determined by X-ray [53] when configurations were also confirmed by ECD comparing their ECD spectra with those previously reported. Urgineanin A was previously reported to have antiproliferative activity at submicromolar concentration against ovarian carcinoma, melanoma and non-small lung cancer cells [31].

Compound **4** is also a racemic mixture and therefore its ECD spectrum could not be interpreted (Figure 4). This result differed from that previously reported for **4** when synthesized from the (3*R*)-3,9-dihydroeucomnalin and wrongly reported as *R* enantiomer [44]. Compound **4,** as well as the starting homoisoflavonoid above described for compound **1**, are a racemic mixture for the presence at C-3 of a proton which could exchange by keto-enol tautomerism.

Racemic natural products are rare and could be obtained from nonenzymatic reactions [54]. However, a chiral tertiary asymmetric carbon in α position to a carbonyl group is easily subject to racemization, as in compounds **1** and **4** [55]. To the best of our knowledge no literatures are available in which compounds **1** and **4** are reported as racemates. However, Sylao et al. 1990 [30] isolated for the first time homoisoflavanoid **1** (named compound **12**) along with other compounds from *Scilla nervosa* subsp. *rigidifolia*. The previously undescribed homoisoflavanoinds were also fully characterized by spectroscopic data (UV, IR, ^1^H and ^13^C NMR and EIMS) but any experiments to determine the absolute configuration of the chiral compounds were neither carried out nor discussed. Among the homoisoflavanoids isolated as new compounds, only for some of them, including **12** (=**1**), was reported the optical specific activity. Based on our experience, these results did not surprise us as the speed of inversion of the configuration of carbon 3, due to the keto-enol tautomerism, could depend on the properties of the solution in which the measurement of the optical rotational power is measured and not from the plant source. Thus, it is possible to have also a scalemic mixture that has optical activity according to the percentage of the predominant enantiomer, as reported for some optical active homoisoflavanois by Sylao et al. 1999 [30]. Scalemic mixtures of two enantiomers are reported in the literature for some different secondary metabolites isolated from several sources as i.e.: phantasmidine, an alkaloid found to be a 4:1 scalemic mixture, enriched in the (2a*R*,4a*S*,9a*S*) enantiomer isolated from the poison frog *Epipedobates anthonyi* [56]; α-pinene, 1-octen-3-ol linalool found as a scalemic mixture of 34% (*R*)-(+) to 66% (*S*)-(−), 95% (*R*)-(−) to 5% (*S*)-(+), 96% (*R*)-(−) to 4% (*S*)-(+) when isolated from the edible wild mushroom *Tricholoma magnivelare* [57]; a furoic acid derivative, containing a chiral center in benzylic position, was found to be a scalemic mixture with an excess of the (*S*) enantiomer, when obtained from the endophytic fungus *Coniothyrium* sp. was isolated from leaves of *Quercus robur* [58]; six pairs of new 6-monosubstituted dihydrobenzophenanthridine alkaloids were separated as scalemic mixtures from the aerial part of *Chelidonium majus*, a plant belonging to the Papaveraceae family, which is widely used in Chinese folk medicine [59].

Compounds **5**, the alkylamide, was isolated for the first time, together with *N*-*trans*-feruloyl octopamine, *N*-*trans*-*p*-coumaroyl octopamine, vanillin, isoscopoletin, ethyl caffeate, ferulic acid and *p*-amminonenzaldehyde, from the eggplant roots [60]. Successively, **5** was also isolated together with close alkylamides from stem parts of *Annona montana* (Annonaceae) and showed significant inhibition of rabbit platelets aggregation induced by thrombin, arachidonic acid, collagen and PAF (platelet-activating factor) and selective cytotoxicity against the P-388 cell line [61,62]. Compound **5** was also found together with other close alkylamides and several phenolic compounds in the methanol extract of basil, lemon thyme, mint, oregano, rosemary, sage and thyme showing antioxidant and anti-inflammatory activities [63].

Alkylamides are a group of bioactive natural compounds widely distributed in plant families and characterized by broad structural variability and a plethora of important biological activities, such as immunomodulatory, antimicrobial, antiviral, larvicidal, insecticidal, diuretic, pungent, analgesic, cannabimimetic and antioxidant activities. Furthermore, they have reinforced the efficacy of antibiotics and inhibited prostaglandin biosynthesis, RNA synthesis and arachidonic acid metabolism [64]. In addition, alkylamides accumulate in rice plants as a defense against the harmful *Cochliobolus miyabeanus* and *Xanthomonas oryzae* pathogens [65].

Flavonoids were also reported to have cytotoxic and anticancer activity, although this aspect was not deeply addressed [66,67]. We found that all tested metabolites were toxic for cancer cells, in a dose- and time-dependent way, although the degree of inhibition of cell viability was cell-type specific. Remarkably, homoisoflavanoids **1**, **3** and **4** were more effective on A431 and HeLa cells compared to immortalized but not transformed HaCaT, thus suggesting that cancer cells were more sensitive to homoisoflavanoid cytotoxicity. The increase of nuclear γH2AX foci upon treatment with metabolites **3** and **4** strongly suggests the occurrence of DNA damage by double-strand breaks (DSBs). However, both **3** and **4** did not increase the production of ROS indicating that a molecular mechanism different from ROS generation is responsible for the observed DNA damage induced by **3** and **4**. Additional experiments are needed to precisely define the molecular mechanism. Moreover, we also detected the signal of cleaved PARP-1 (89 kDa) by immunoblot while the level of the cell cycle inhibitor p21WAF, which causes cell growth arrest preventing the induction of apoptosis, was reduced, thus confirming that the decrease of cell viability was due to cell death rather than cell cycle arrest.

Isolated compounds displayed additional interesting biological properties. As previously described but isolated for the first time in a *Crinum* species, **5** is a strong anti-α-glucosidase and anti-α-amylase inhibitor. Recently, the efficacy of flavanoids for type 2 diabetes mellitus (T2DM) was shown in clinical therapies. T2DM is a metabolic disorder associated with the overproduction of free radicals and oxidative stress. Diabetes is increasing exponentially, and the World Health Organization estimates that by the year 2030, it could be the seventh cause of death worldwide [68,69]. The flavonoids appear to play a role in multiple processes involved in T2DM [70,71] such as the regulation of glucose metabolism, hepatic enzymes activities and a lipid profile [72]; thus, studies on nutritional flavonoids to manage diabetes and its complications are currently in progress [73].

Interestingly, the *N*-*p*-coumaroyltyramine alkylamide (**5**) was selectively cytotoxic against A431 and HeLa cancer cells while it protected immortalized HaCaT cells against oxidative stress induced by hydrogen peroxide. This result is highly relevant for a potential application of **5** in anticancer therapy. Furthermore, we observed the antidiabetic properties of **5** and the anti-acetylcholinesterase activity in compounds **1**–**4**. Homoisoflavanoids are also known for their anti-acetylcholinesterase properties, a key enzyme to Alzheimer’s disease development [74]. We detected anti-acetylcholinesterase activity in compound **3**, a property that had not yet been reported for urgineanins A and B, to our knowledge. Finally, as we recently showed that *Crinum jagus* contained antiviral compounds, we measured the antiretroviral activity of isolated compounds. We report that all compounds possessed anti-retroviral potential, **3** and **4** being the most potent inhibitors.

## 5. Conclusions

Four homoisoflavanoids and one alkylamide were isolated and characterized from *C. biflorum*, an Amaryllidaceae plant used in African traditional medicine, collected in Senegal. Flavonoids **1**, **3** and **4** showed promising anticancer properties being cytotoxic at low micromolar concentrations towards HeLa and A431 human cancer cell lines. The *N*-*p*-coumaroyltyramine (**5**) was selectively toxic to A431 and HeLa cancer cells, while it protected immortalized HaCaT cells against oxidative stress induced by hydrogen peroxide. Compounds **1**–**4** also inhibited acetylcholinesterase activity, with compound **3** being the most potent. The anti-amylase and the strong anti-glucosidase activity of compound **5** were confirmed. This study extends the chemical library of compounds that can be a potential candidate for the treatment of cancer, viral infections, diabetes and Alzheimer’s disease.

## Figures and Tables

**Table 1:** Cytotoxic activities of metabolites **1**–**5**. Compounds IC_50_ (μM) ^1^.

Compound	HaCaT	A431	HeLa
**1**	>10	6.0	1.0
**2**	>10	>10	4.0
**3**	>10	4.0	4.5
**4**	>10	6.5	5.5
**5**	Not detected	>10	7.5

^1^ IC_50_ was calculated after 24 h of incubation.
